# Effect of ice slushy ingestion and cold water immersion on thermoregulatory behavior

**DOI:** 10.1371/journal.pone.0212966

**Published:** 2019-02-27

**Authors:** Hui C. Choo, Jeremiah J. Peiffer, João P. Lopes-Silva, Ricardo N. O. Mesquita, Tatsuro Amano, Narihiko Kondo, Chris R. Abbiss

**Affiliations:** 1 Centre for Exercise and Sports Science Research, School of Medical and Health Sciences, Edith Cowan University, Joondalup, Western Australia, Australia; 2 School of Psychology and Exercise Science, Murdoch University, Murdoch, Western, Australia, Australia; 3 School of Physical Education and Sport, University of São Paulo (USP), São Paulo, São Paulo, Brazil; 4 Faculty of Education, Niigata University, Niigata, Niigata Prefecture, Japan; 5 Laboratory for Applied Human Physiology, Graduate School of Human Development and Environment, Kobe University, Kobe, Hyōgo Prefecture, Japan; Nottingham Trent University, UNITED KINGDOM

## Abstract

Two studies were conducted to examine the effects of ice slushy ingestion (ICE) and cold water immersion (CWI) on thermoregulatory and sweat responses during constant (study 1) and self-paced (study 2) exercise. In study 1, 11 men cycled at 40–50% of peak aerobic power for 60 min (33.2 ± 0.3°C, 45.9 ± 0.5% relative humidity, RH). In study 2, 11 men cycled for 60 min at perceived exertion (RPE) equivalent to 15 (33.9 ± 0.2°C and 42.5 ± 3.9%RH). In both studies, each trial was preceded by 30 min of CWI (~22°C), ICE or no cooling (CON). Rectal temperature (T_re_), skin temperature (T_sk_), thermal sensation, and sweat responses were measured. In study 1, ICE decreased T_re-_T_sk_ gradient versus CON (p = 0.005) during first 5 min of exercise, while CWI increased T_re-_T_sk_ gradient versus CON and ICE for up to 20 min during the exercise (p<0.05). In study 2, thermal sensation was lower in CWI versus CON and ICE for up to 35–40 min during the exercise (p<0.05). ICE reduced thermal sensation versus CON during the first 20 min of exercise (p<0.05). In study 2, CWI improved mean power output (MPO) by ~8 W, compared with CON only (p = 0.024). In both studies, CWI (p<0.001) and ICE (p = 0.019) delayed sweating by 1–5 min but did not change the body temperature sweating threshold, compared with CON (both p>0.05). Increased T_re_-T_sk_ gradient by CWI improved MPO while ICE reduced T_re_ but did not confer any ergogenic effect. Both precooling treatments attenuated the thermal efferent signals until a specific body temperature threshold was reached.

## Introduction

During self-paced exercise with increased exogenous heat load, behavioral thermoregulation can be achieved through adjusting the work rate to manipulate metabolic heat production since heat dissipation is limited by involuntary mechanisms, i.e., sweating and cutaneous vasodilation [1]. Under this paradigm, skin and core temperature and thermal perception have been identified as controllers of thermoregulatory behavior [2–4]. These controllers may modify thermoregulatory behavior independently [3, 5]. Indeed, improved thermal sensation and greater work output have been observed following oral L-menthol mouth rinse [5], face cooling via fan or application of topical menthol gel [3], without changes in the skin and core temperature.

External cooling via whole body cold water immersion (CWI) and internal cooling via ingestion of ice slushy (ICE) have often been used as ergogenic aids before exercise in the heat [6]. The aim of precooling is to create a greater heat sink for subsequent metabolic heat production; however, the ergogenic mechanisms underlying precooling may be specific to different cooling methods. For example, whole body CWI involves direct contact with a large body surface area and thus resulting in reduced skin blood flow and increased core-to-skin temperature gradient, whereas ICE has a more direct effect on the core body temperature [7]. Recent meta-analyses have shown that CWI improves aerobic exercise performance in warm environments [6], with a lesser beneficial effect observed in moderate conditions (i.e., ambient temperatures between 18–26°C) [8]. During exercise in temperate environments, drastic muscle cooling during CWI has been shown to negatively impact delivery of oxygen (O_2_) and substrates to locomotive muscle as assessed by near-infrared spectroscopy (NIRS), resulting in increased anaerobic metabolism during subsequent exercise in temperate environments [9, 10]. It is worth noting that exercise in the heat has also been shown to impair muscle blood volume and tissue oxygenation assessed by NIRS [11].

ICE is logistically less challenging than CWI and has minimal muscle cooling effect, making this cooling method preferable to CWI. However, a recent meta-analysis showed that CWI and ICE may elicit different influences on the thermoregulatory behavior during exercise in the heat via changes in skin and core temperatures and thermal perception [6]. Specifically, while both ICE and CWI effectively reduced core temperature, but only CWI had a clear beneficial effect on exercise performance concomitant with reductions in skin temperature and thermal sensation. Additional benefits of CWI versus ICE include a greater body surface area being directly cooled during whole body immersion, a continued cooling effect after immersion [12], the practicality of ICE being limited by an optimal drinking volume required for significant core body cooling effect [13], and improved whole body fluid balance through lesser sweat loss [6]. Sweating during exercise is initiated by both thermal and non-thermal factors, with nitric oxide being identified as a non-neural regulating factor [14, 15]. ICE has been shown to activate the intra-abdominal thermoreceptors but minimally affect skin temperature and skin blood flow [16, 17]. Hence, sweating during ICE is predominantly regulated by thermal reflexes, whereas direct skin cooling during CWI is likely to regulate the sweat response via activation of the thermoreceptors and the nitric oxide pathway through blood flow reduction.

The present study aimed to examine the differences in some thermoregulatory parameters (i.e., core-to-skin temperature gradient, sweat response and thermal sensation) and muscle perfusion (NIRS parameters) during CWI and ICE versus a no cooling control condition (CON), and the consequential influence on thermoregulatory behavior as indicated by total work output during exercise in the heat. Accordingly, two studies were conducted. In the first study, the thermoregulatory parameters and muscle perfusion were assessed during 60 min of exercise at a fixed intensity following CWI, ICE and CON. In the second study, thermoregulatory behavior was investigated during 60 min of cycling at a fixed rating of perceived exertion (RPE). The RPE clamp protocol allows individuals to modify power output continually based on the regulatory role of perceived exertion in behavioral thermoregulation [18], and has been utilised to investigate the differential influences of thermal perception and temperature on thermoregulatory behavior [3, 5]. In the first study, it was hypothesized that CWI would result in a greater core-to-skin temperature gradient, but would attenuate the sweat responses and muscle perfusion when compared with ICE and CON. In the second study, we hypothesized CWI would improve mean power output and total work output relative to CON and ICE.

## Materials and methods

### Participants

The experimental procedures were approved by Edith Cowan University ethics committee for human research and were conducted according to the principles expressed in the Declaration of Helsinki. All participants were recruited locally via the institutional intranet portal and social media. All procedures and associated risks were made known to the participants before obtaining signed consent. For study 1, *a priori* analysis using whole body sweat loss data from a previous study [19] was performed using the calculated effect size of 0.78, an α of 0.05, and a β of 0.2. Minimum of 7 participants were required to identify significant difference in whole body sweat loss between conditions. Nevertheless, 13 men were recruited for study 1; however, only 11 men completed all trials (mean ± SD; age: 27 ± 6 y, body mass: 77.5 ± 10.5 kg, height: 177.1 ± 7.9 cm, sum of 4 skinfolds: 58.1 ± 25.7 mm, Peak O_2_ uptake (${\dot{V}O}_{2\mathrm{peak}}$): 43.9 ± 11.4 mL·kg^-1^·min^-1^, peak aerobic power: 288 ± 64 W). One participant withdrew from the study due to personal reasons and another could not complete the exercise task. For study 2, power analysis was determined using an effect size of 0.80 based the work output data from a previous study using similar RPE clamp protocol [3]. A minimum of 7 participants were required to identify significant difference between conditions with an α of 0.05 and a β of 0.2. Eleven out of 13 participants completed the study (mean ± SD; age: 30 ± 6 y, body mass: 80.6 ± 12.8 kg, height: 1.8 ± 0.1 m, ${\dot{V}O}_{2\mathrm{peak}}$: 51.1 ± 8.2 mL.kg^-1^.min^-1^, sum of 7 skinfold: 131.2 ± 52.6 mm). Two participants did not complete the trials due to personal reasons. Thirteen participants were recruited for each study after taking into consideration attrition, missing data and inherent differences in the study designs. All participants were recreationally active who engaged in physical activity (e.g., cycling, running and soccer) for ≥3 times per week within the past two years, non-smokers and free from cardiovascular disease. All participants completed a preliminary visit and three experimental trials. Participants were asked to: 1) avoid strenuous exercise and alcohol consumption during the 24 h before each trial; 2) avoid caffeine during the 12 h before each trial; and 3) keep their diet, physical activity and sleep habits consistent before each trial, assisted by a 1-d dietary intake and physical activity record.

### Preliminary measurements

Anthropometric measurements and ${\dot{V}O}_{2\mathrm{peak}}$ were assessed during a preliminary session. The ${\dot{V}O}_{2\mathrm{peak}}$, defined as the highest 30-sec average, was assessed by an incremental cycling exercise test (Velotron Racermate, Seattle, WA, USA) starting at 70 W and increased by 35 W·min^-1^ until cadence dropped below 60 rpm. Minute ventilation, carbon dioxide production and ${\dot{V}O}_{2}$ was measured by a calibrated metabolic cart (TrueOne 2400, ParvoMedics, Utah, USA) during the incremental exercise test. Peak power output was prorated from the last completed stage plus the time in the last uncompleted stage multiplied by 35 W [20]. Heart rate ([HR], Polar S810i, Polar Electro Oy, Kempele, Finland) and RPE [21] were also assessed during the exercise. In study 2, participants performed a standardised familiarisation trial adapted from Lander et al. [22] after the incremental exercise test. The familiarisation trial began at RPE 11 for 4 min and increased to RPE 13 (3 min), RPE 15 (2 min) and ended at RPE 19 (1 min).

### Experimental trials

In both studies, participants completed three experimental trials separated by at least 48 h, and the time of trials was kept within 2 h between sessions for each participant. All participants arrived 30 min before the experimental trials. Following urine specific gravity index (USG, Atago hand-held refractometer, Model Master-URC/Nα, Tokyo, Japan), nude body mass measurement (Model ID1, Mettler Toledo, Columbus, Ohio, USA) and self-insertion of a rectal thermistor, participants proceeded to a regulated climate chamber for at least 20 min for thermal equalisation and further instrumentation

In study 1, participants performed one of the three 30-min pre-exercise treatments in a randomized crossover manner: 1) ingestion of 1.25 g·kg^-1^·5 min^-1^ of an ice slushy mixture (-0.7 ± 0.1°C) with added orange flavoured syrup (Cottee’s Foods, NSW, Australia); 2) mid-sternal level CWI at 22.1 ± 0.1°C; and 3) passive rest on a chair beside the cycle ergometer during CON. During CWI and CON, participants consumed 1.25 g·kg^-1^·5 min^-1^ of warm fluid (36.3 ± 0.6°C) with added orange flavoured syrup. For all conditions, the mixed drinks contained 6% carbohydrate. At 9 min 9 sec ± 1 min 31 sec after the end of cooling, participants cycled for 60 min at 40–50% of peak aerobic power at 33.2 ± 0.3°C, 45.9 ± 0.5%RH. In study 2, participant completed three trials which involved 30 min of pre-exercise treatments. Before exercise, they consumed 1.25 g·kg^-1^·5 min^-1^ of ICE (0.1 ± 0.1°C) containing 0% carbohydrate, completed 30 min of CWI at 22.3 ± 0.2°C, and passive rest (CON) in a randomized, crossover manner. Warm water (35.8 ± 0.3°C) was consumed at 1.25 g·kg^-1^·5 min^-1^ during CWI and CON. For both studies, the ice slushy mixture (1:1 mixture of ice and liquid) was prepared using a commercially available food blender. All drinks consumed during precooling and exercise were stored in an insulated flask, and temperature of the drinks was measured immediately before serving.

In study 2, the experimental trials required participants to cycle for 60 min at a pace equivalent to 15 or ‘hard or heavy’ on the 15-point RPE scale [21] at 33.9 ± 0.2°C and 42.5 ± 3.9%RH. Participants had access to the elapsed time, but no other feedback or verbal encouragement was given. Exercise commenced at 10 min 22 sec ± 1 min 4 sec after the end of cooling. In both studies, thermal sensation [23] and RPE [21] were assessed every 5 min, and water consumption during the exercise was matched between trials for each participant.

### Temperature and sweat measurements

Temperature and sweat data were logged at 0.2 Hz continuously during the experimental trials by a Squirrel data logger (Model 2040, Grant Instruments Ltd., Cambridge, UK). Weighted mean skin temperature was calculated from the measurement of four sites [24] over the sternum (T_st_), forearm (T_arm_), thigh (T_th_) and calf (T_ca_) (YSI 409B thermistors, Dayton, OH, USA). Rectal temperature (T_re_) was measured via a thermistor (Monatherm Thermistor 400 Series, Mallinckrodt Medical, St. Louis, MO, USA) self-inserted 10 cm past the anal sphincter. Mean body temperature (T_b_) was calculated as (0.79 × T_re_) + (0.21 × T_sk_) [25].

Local sweat rate ([LSR], mg·cm^-2^·min^-1^) was measured using ventilated sweat capsules (5.31 cm^2^) attached to the left dorsal forearm 5 cm below the antecubital fossa (LSR_arm_), and the left thigh 15 cm above the superior border of the patella (LSR_th_). Dry air ventilated the capsules at 1.5 mL·min^-1^ and the water content of the effluent air was measured using capacitance hygrometers (HMP60, Vaisala, Helsinki, Finland). Onset of sweating in terms of exercise time was determined by fitting the 1-min averaged data using a one-phase exponential association model [26]. Thermoregulatory sweating threshold and sensitivity were determined by plotting LSR averaged from both sites against T_re_ and T_b_ as previously described [27]. Whole body sweat loss was determined from the change in body mass to the nearest 10 g, taking into consideration the total volume of fluid consumed.

### NIRS measurements

A near-infrared spectroscopy system (Niromonitor NIRO-200, Hamamatsu Photonics, Japan) was used to measure tissue chromophore concentration changes in the right vastus lateralis at 10 Hz (Powerlab 16/30 ML 880/P, ADInstruments, New South Wales, Australia). The NIRS probe, with an interoptode distance of 4 cm, was secured to the right thigh with the centre of the probe 15 cm above the superior border of the patella towards the greater trochanter. A black polyethylene sheet (7 cm × 5 cm × 30 μm) was secured over the NIRS probe to minimise ambient light contamination, and participants placed their legs in a polyethylene bag (365 cm × 228 cm × 30 μm) to protect the measuring instruments from water damage during CWI.

Using the modified Beer-Lambert method, the NIRS system measures changes (μM·cm^-1^) in oxyhaemoglobin (OxyHb), deoxyhaemoglobin (Hb) and total haemoglobin (tHb) at 775, 810 and 850 nm from an arbitrary initial value. Simultaneously, tissue oxygenation index (TOI, %) is determined based on the spatially resolved spectroscopy method. Changes in the tHb and TOI reflect muscle blood volume and ratio between oxygenated haemoglobin and total haemoglobin, respectively [28]. The NIRS data were averaged over 1 min and expressed as absolute differences from the baseline values.

### Heart rate, mean arterial pressure and skin perfusion

HR was recorded continuously during the experimental trials using the same Polar telemetric monitor. In study 1, skin perfusion was measured at the left forearm by laser Doppler flowmetry system (PeriFlux 5000 with thermostatic probe 457, Perimed AB, Järfälla, Stockholm, Sweden), expressed as arbitrary perfusion units (PU). However, due to probe displacement during the exercise, the authors decided not to include the PU signals in the data analysis. In study 2, skin PU was measured at the left thigh. Distance between the sweat capsule, skin thermistor and the laser Doppler probe were kept at 2 cm. Mean arterial pressure (MAP) was obtained from the blood pressure waveform recorded from a finger (Finapres NOVA, Finapres Medical Systems©, Amsterdam, The Netherlands). The PU and MAP signals were sampled at 10 Hz using the same data acquisition software as the NIRS signals. MAP was not recorded during the RPE clamp exercise (study 2) to minimise distraction to the participants. Cutaneous vascular conductance (CVC) was determined using the quotient between PU and MAP at baseline and at the end of cooling.

### Statistical analysis

Statistical analyses were performed using the nlme and emmeans packages for R v3.5.0 (R Core Team, R Foundation for Statistical Computing, Vienna, Austria). Linear mixed effects modeling allowed inclusion of data sets with missing values during the exercise for T_re_ and the NIRS signals due to thermistor displacement and probe damage, respectively. The dependent variables were analyzed in separate linear mixed models fitted with restricted maximum likelihood. The initial models included a by-participant random intercept. Random effects for intercept and for slope with regards to condition, and covariance between intercepts and slopes were incorporated where indicated by minimising the values for Akaike information criteria. Marginal p-values were reported. Bonferroni correction was applied for multiple pairwise comparisons at a given time point. Significance level was accepted at p≤0.05. A total of 19 data sets from study 1 and 2 were included in the analysis of the sweating threshold and sensitivity. One participant partook in both studies and thus his data set was included only once. Additionally, two data sets from study 1 were excluded due to water damage during CWI and due to missing values >30 min during the exercise. All data are expressed as mean ± SD.

## Results

### Study 1

#### Baseline body mass and USG, physiological and perceptual responses during exercise

A significant effect was present for baseline body mass (p = 0.006) with post-hoc analysis revealing that baseline body mass was lower in CON versus ICE (p = 0.008), but no difference was observed between CWI and CON (p = 0.195) or between CWI and ICE (p = 0.979, CON: 77.3 ± 9.7 kg, CWI: 77.6 ± 10.2 kg, ICE: 77.8 ± 9.9 kg). USG before the exercise was similar between conditions (p = 0.494, CON: 1.013 ± 0.006 g·ml^-1^, CWI: 1.010 ± 0.007 g·ml^-1^_,_ ICE: 1.011 ± 0.008 g·ml^-1^). Table 1 shows the mean physiological and perceptual responses during the 60 min of exercise. Whole body sweat loss was significantly lower in CWI compared with CON (p = 0.014) while there was no difference between ICE and CON (p = 0.161). Mean HR was lower in CWI relative to CON (p = 0.025), while differences between CWI and ICE did not reach statistical significance (p = 0.068). Thermal sensation, RPE, LSR_arm_ and LSR_th_ were not significantly different between conditions (Table 1).

**Table 1:** Mean physiological and perceptual responses during 60 min of cycling at fixed exercise intensity (study 1), and during the RPE clamp exercise (study 2).

	CON	CWI	ICE	P-value
**Study 1:**				
Whole body sweat loss (mL)	1244 ± 374*	1064 ± 343	1128 ± 329	0.016
Thermal sensation (AU)	6.0 ± 1.1	5.7 ± 0.6	5.8 ± 0.7	0.384
RPE (AU)	13.5 ± 1.9	13.4 ± 1.8	13.4 ± 1.6	0.982
Heart rate (beats∙min^-1^)	154 ± 14*	149 ± 15	153 ± 14	0.018
T_re_Δ (°C) (*n* = 8)	1.3 ± 0.5^#^	1.2 ± 0.6^#^	1.6 ± 0.6	<0.001
LSR_arm_	1.25 ± 0.75	1.10 ± 0.53	1.14 ± 0.44	0.470
LSR_th_	0.71 ± 0.46	0.74 ± 0.36	0.69 ± 0.35	0.772
**Study 2:**				
Mean power output (W)	130 ± 20*	138 ± 18	129 ± 25	0.018
Total work output (kJ)	470 ± 74*	498 ± 65	464 ± 90	0.018
Whole body sweat loss (mL)	1394 ± 381*	1239 ± 367	1396 ± 119*	0.004
Heart rate (beats∙min^-1^)	144 ± 20	141 ± 14	144 ± 15	0.679
T_re_Δ (°C)	1.4 ± 0.5^#^	1.2 ± 0.6^#^	1.7 ± 0.5	<0.001
LSR_arm_	1.21 ± 0.35	1.21 ± 0.53	1.20 ± 0.60	0.994
LSR_th_	0.63 ± 0.19	0.68 ± 0.29	0.66 ± 0.27	0.592

CON, control; CWI, cold water immersion, ICE, ice slushy ingestion; RPE, rating of perceived exertion; T_re_Δ, magnitude of increase in rectal temperature during exercise; LSR_arm_, local sweat rate for the arm at the end of exercise; LSR_th_, local sweat rate for the thigh at the end of exercise

* p<0.05 versus CWI

^#^ p<0.05 versus ICE.

Data are mean ± SD for *n* = 11 unless otherwise stated. See S5 Table for effect sizes (Cohen’s *d*) calculated from mean differences between conditions and pooled SD.

#### T_sk_, T_re_ and T_re_-T_sk_ gradient

**Fig 1:**
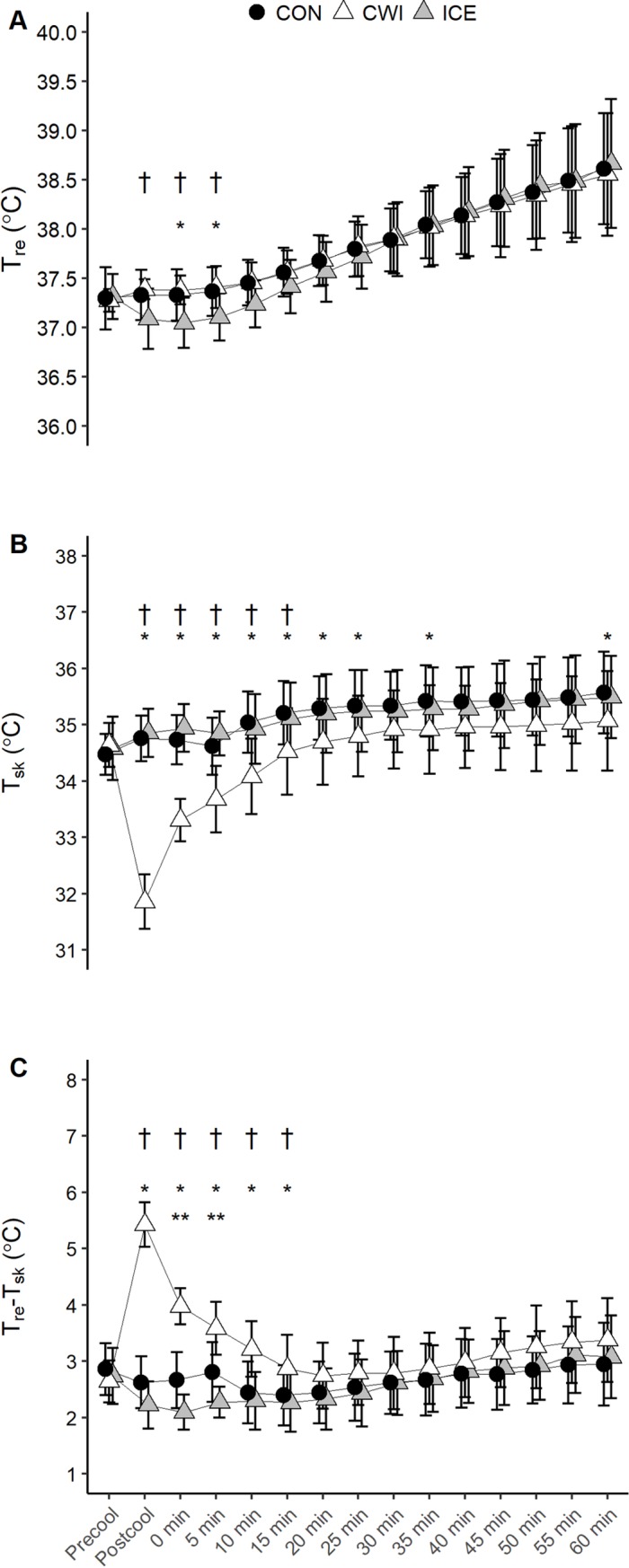
**T_re_ (A), T_sk_ (B), and T_re_-T_sk_ gradient (C) during 60 min of cycling at fixed intensity (study 1).** CON, control; CWI, cold water immersion; ICE, ice slushy ingestion; * p<0.05 CWI versus CON; ** p<0.05 ICE versus CON; † p<0.05 CWI versus ICE. Data are mean ± SD for *n* = 11 for T_sk_. Due to missing data at certain time points during the exercise, data for T_re_ and T_re_-T_sk_ gradient are *n* = 10 during the first 10 min of exercise for all conditions and *n* = 8 or 9 thereafter (see S1 and S2 Tables for clarification).

One participant’s data for T_re_ were removed from the analysis due to water damage during CWI. A significant interaction effect was observed for T_re_ (p = 0.020). ICE decreased T_re_ by 0.3°C during the first 5 min of exercise when compared with CWI and CON (p<0.05, Fig 1A). However, the magnitude of increase in T_re_Δ during the exercise was higher in ICE relative to CON (p = 0.012) and CWI (p = 0.001, Table 1), resulting in similar T_re_ at the end of exercise (Fig 1). An interaction effect was observed for T_sk_ (p<0.001) such that it was significantly lower in CWI compared with ICE and CON during the exercise (Fig 1B). T_re_-T_sk_ gradient exhibited an interaction effect (p<0.001) such that CWI increased the gradient during the first 15 min of exercise compared with CON and ICE (p<0.05, Fig 1C). ICE decreased T_re_-T_sk_ gradient relative to CON during the first 5 min of exercise (p<0.05).

#### NIRS data

**Fig 2:**
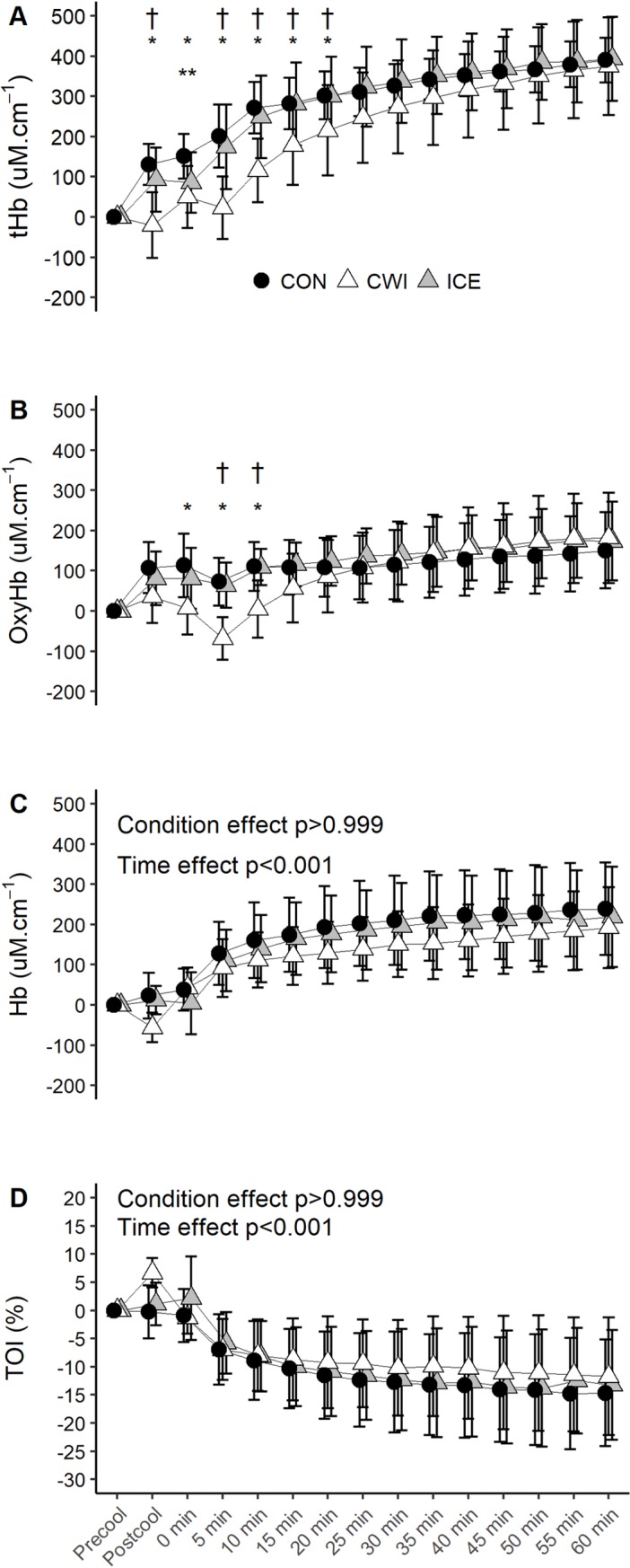
**Changes in tHb (A), HbO_2_ (B), Hb (C) and TOI (D) during 60 min of cycling at fixed intensity (study 1).** CON, control; CWI, cold water immersion; ICE, ice slushy ingestion; * p<0.05 CWI versus CON; ** p<0.05 ICE versus CON; † p<0.05 CWI versus ICE. Data are expressed as absolute changes from the baseline values and are mean ± SD for *n* = 11, except for the final 20 min of exercise during CON and the final 10 min of exercise during ICE where *n* = 10 due to probe damage.

An interaction effect was observed for tHb (p<0.001) whereby CWI significantly decreased tHb relative to CON and ICE during the first 20 min of exercise (Fig 2A). OxyHb exhibited an interaction effect (p<0.001) such that it was lower in CWI versus CON and ICE during the initial 10 min of exercise (p<0.05, Fig 2B). Hb showed an effect for time (p<0.001), but no main condition (p>0.05) or interaction effect (p = 0.350, Fig 2C) was observed. Although TOI demonstrated an effect for time (p<0.001), there was no condition (p>0.05) or interaction effect (p = 0.580, Fig 2D).

### Study 2

#### Baseline body mass and USG

There was no difference between conditions for baseline body mass (p = 0.498, CON: 80.2 ± 13.3 kg, CWI: 80.5 ± 12.9 kg, ICE: 80.3 ± 12.9 kg) and USG (p = 0.255, CON: 1.016 ± 0.009 g·ml^-1^, CWI: 1.017± 0.007 g·ml^-1^_,_ ICE: 1.013 ± 0.008 g·ml^-1^). Four participants took up to 34 min 32 sec to ingest the given volume in the ICE trials.

#### MPO, total work output, HR and sweat responses

MPO in CWI was greater than CON (p = 0.024), but there was no difference between CON and ICE (p>0.999) or between CWI and ICE (p = 0.263, Table 1). Similarly, total work output in CWI was greater than CON (p = 0.024), whereas there was no difference between CON and ICE (p>0.999) or between CWI and ICE (p = 0.263, Table 1). There was no condition effect for the mean HR response during the exercise (p = 0.679, Table 1). CWI decreased whole body sweat loss relative to CON (p = 0.012) and ICE (p = 0.011); however, there was no condition effect for LSR_arm_ (p = 0.994) or LSR_th_ (p = 0.592).

#### T_sk_, T_re_, T_re_-T_sk_ gradient and thermal sensation

**Fig 3:**
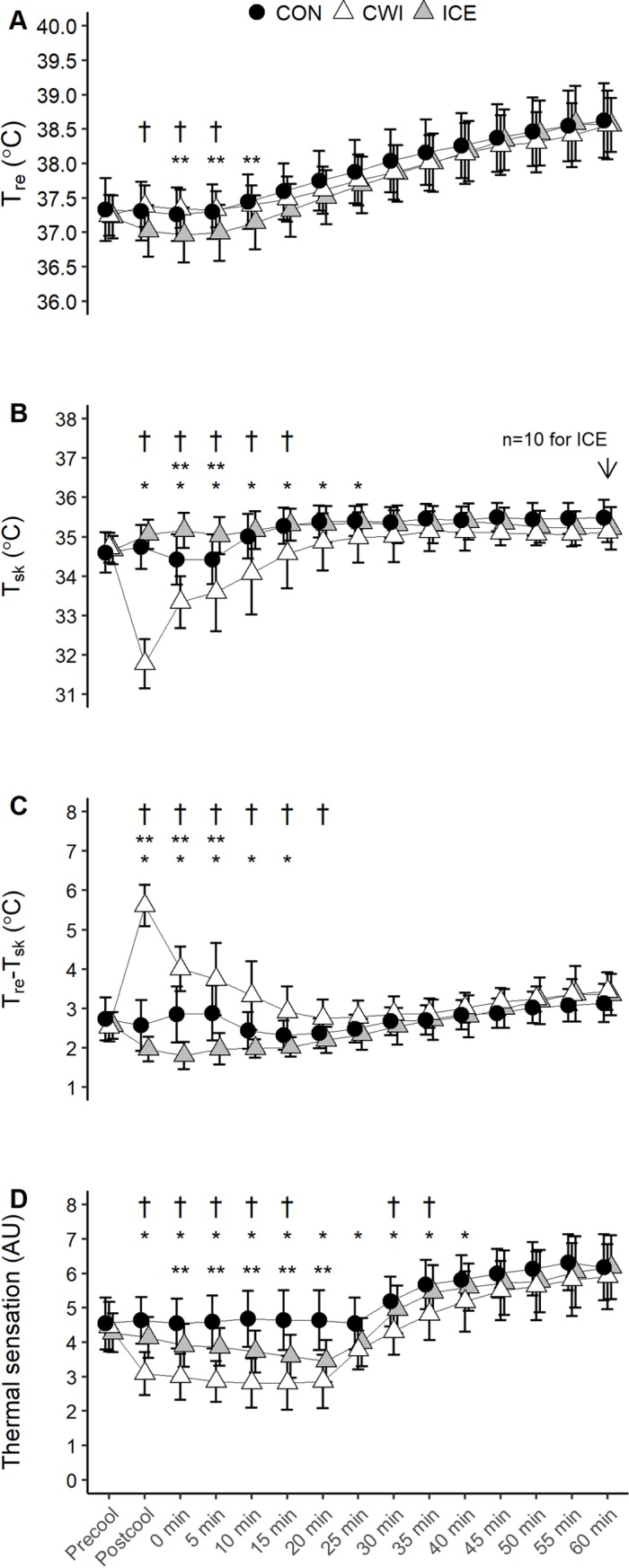
**T_re_ (A), T_sk_ (B), and T_re_-T_sk_ gradient (C), and thermal sensation (D) during 60 min of cycling at RPE 15 (study 2).** CON, control; CWI, cold -water immersion; ICE, ice slushy ingestion; * p<0.05 CWI versus CON; ** p<0.05 ICE versus CON; † p<0.05 CWI versus ICE. Data are mean ± SD for *n* = 11 unless otherwise stated. Due to missing data at certain time points during the exercise, data for T_re_ and T_re_-T_sk_ gradient are *n* = 11 during the first 25 min of exercise for all conditions, and *n* = 10 for CWI and ICE thereafter (see S3 and S4 Tables for clarification).

Significant interaction effect was observed for T_re_ (p = 0.003) such that it was lower by ~0.3°C in ICE versus CWI and CON during the first 5–10 min of exercise (Fig 3A). However, the magnitude of increase in T_re_ during the 60 min of exercise was greatest in ICE compared with CON (p = 0.003) and CWI (p<0.001, Table 1). An interaction effect was observed for T_sk_ (p<0.001). CWI decreased T_sk_ for up to 15–20 min during the exercise compared with CON and ICE (p<0.05, Fig 3B), while ICE increased T_sk_ during the first 5 min of exercise compared with CON (p<0.05). T_re_-T_sk_ gradient depicted an interaction effect (p<0.001) whereby it increased during the first 15–20 min of exercise in CWI relative to CON and ICE (p<0.05, Fig 3C). ICE decreased T_re_-T_sk_ gradient during the first 5 min of exercise compared with CON (p<0.05). An interaction effect was observed for thermal sensation (p<0.001). Both CWI and ICE reduced thermal sensation during the exercise compared with CON (p<0.05), but final values were not different between conditions (p>0.05, Fig 3D). CWI decreased thermal sensation during the first 15 min of exercise and between 30 to 35 min during the exercise, when compared with ICE (p<0.05).

#### NIRS data and skin PU

**Fig 4:**
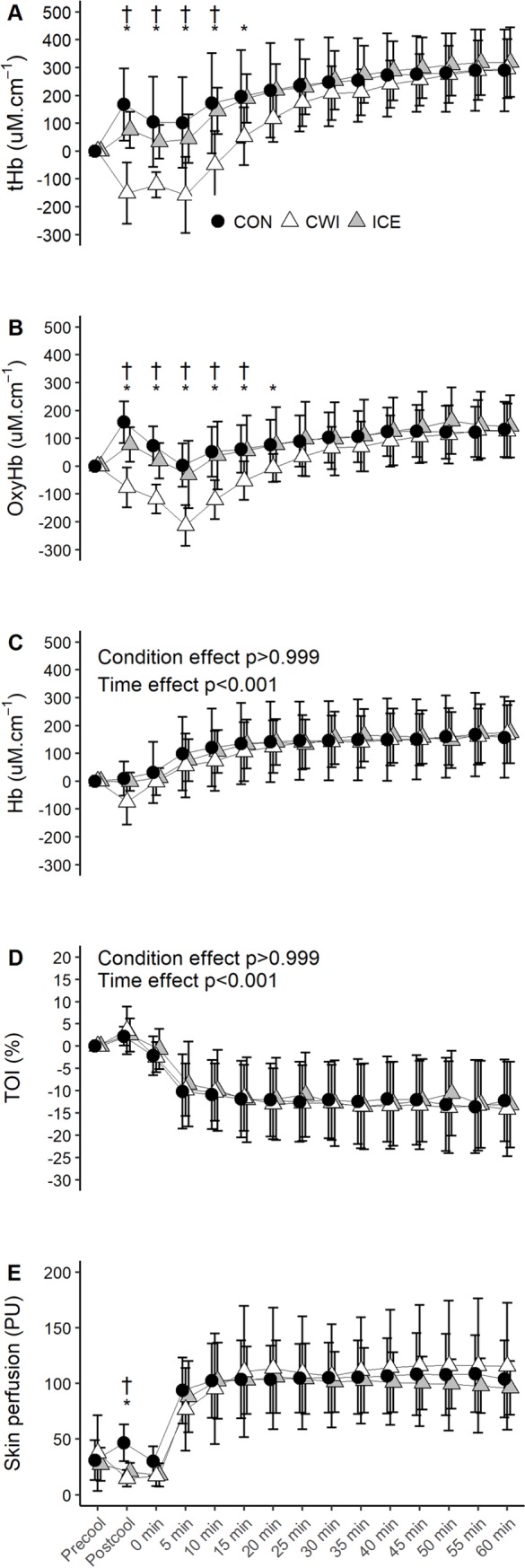
**Changes in tHb (A), OxyHb (B), Hb (C) TOI (D), and skin PU (E) during 60 min of cycling at RPE 15 (study 2).** CON, control; CWI, cold water immersion; ICE, ice slushy ingestion; * p<0.05 CWI versus CON; ** p<0.05 ICE versus CON; † p<0.05 CWI versus ICE. Data are expressed as absolute changes from the baseline values and are mean ± SD for *n* = 11, except for the final 30 min of exercise during CWI where *n* = 10 due to probe damage.

An interaction effect was evident for tHb (p<0.001) in which it decreased during CWI and for up to 10–15 min during the exercise compared with CON and ICE (p<0.05, Fig 4A). Significant interaction effect was observed for OxyHb (p = 0.005) whereby it decreased during CWI for up to 15–20 min during the exercise compared with CON and ICE (p<0.05, Fig 4B). OxyHb was also lower at the end of cooling during ICE compared with CON (p = 0.034). Hb increased during the exercise (p<0.001) but there was no main condition (p>0.999) or interaction effect (p = 0.546, Fig 4C). No effect for condition (p>0.05) or interaction (p>0.05) was observed for TOI, although it decreased during the exercise (p<0.001, Fig 4D).

Skin PU depicted an interaction effect (p = 0.005) whereby both CWI (p = 0.012) and ICE (p = 0.044) decreased PU by the end of cooling relative to CON (Fig 4E). An interaction effect was evident for CVC (p = 0.001). CVC was not different between conditions at baseline (p>0.05, CON: 0.39 ± 0.17 PU·mmHg^-1^, CWI: 0.49 ± 0.40 PU·mmHg^-1^, ICE: 0.37 ± 0.23 PU·mmHg^-1^). At the end of cooling, CWI (p<0.001) and ICE (p = 0.001) decreased CVC relative to CON (CON: 0.54 ± 0.20 PU·mmHg^-1^, CWI: 0.19 ± 0.11 PU·mmHg^-1^, ICE: 0.22 ± 0.10 PU·mmHg^-1^). When expressed as percentage of the baseline values, the changes in CVC were +15%, -61% and -40% for CON, CWI and ICE, respectively.

### Sweat threshold and sweat sensitivity from studies 1 and 2

CWI (p<0.001) and ICE (p = 0.019) delayed sweat recruitment in terms of exercise time compared with CON (Table 2). Additionally, CWI delayed sweating onset by ~4 min relative to ICE (p<0.001). Sweating occurred at a lower T_re_ in ICE versus CON (p = 0.007) and CWI (p<0.001), while CWI resulted in an elevated T_re_ threshold for sweating compared with CON (p = 0.007, Table 2). However, there was no difference between conditions for the T_b_ threshold (p = 0.973). T_re_ sweat sensitivity was increased following CWI compared with CON (p = 0.007) and ICE (p = 0.009). There was a significant effect for T_b_ sweat sensitivity (Table 2). CWI resulted in a higher sweat sensitivity compared with CON (p = 0.037), while the difference between CON and ICE did not reach statistical significance (p = 0.107).

**Table 2:** T_re_ and T_b_ at the onset of sweating and slopes of regression lines determined after plotting average LSR against T_re_ and T_b_ during exercise at fixed intensity (study 1) and during the RPE clamp exercise (study 2).

	CON	CWI	ICE	P-value
Onset of sweating (min)	1.8 ± 1.8* ^#^	6.4 ± 2.2^#^	2.7 ± 1.6*	<0.001
T_re_ sweat threshold (°C)	37.0 ± 0.3* ^#^	37.1 ± 0.2^#^	36.8 ± 0.3*	<0.001
T_b_ sweat threshold (°C)	36.5 ± 0.3	36.5 ± 0.3	36.5 ± 0.4	0.973
T_re_ sweat sensitivity (mg∙cm^-2^∙min^-1^∙°C^-1^)	1.51 ± 0.61*	1.89 ± 0.82	1.42 ± 0.68*	0.001
T_b_ sweat sensitivity (mg∙cm^-2^∙min^-1^∙°C^-1^)	1.18 ± 0.42*	1.36 ± 0.55	1.39 ± 0.63	0.007

CON, control; CWI, cold water immersion; ICE, ice slushy ingestion; T_re_, rectal temperature; T_b_, weighted mean body; LSR, average local sweat rate for arm and thigh

* p<0.05 versus CWI

^#^ p<0.05 versus ICE. Data are mean ± SD for *n* = 19 from study 1 and 2. See S5 Table for effect sizes (Cohen’s *d*) calculated from mean differences between conditions and pooled SD.

## Discussion

The present study compared the effects of precooling internally via ICE and externally via CWI on thermoregulatory responses (e.g., T_sk_ and T_re_) during steady state exercise (study 1) and thermoregulatory behavior (i.e., power output and total work output) during the RPE clamp protocol (study 2). These cooling methods were utilised for their distinct influences on T_sk_ and T_re_. As hypothesised, study 1 demonstrated that ICE resulted in a narrower T_re_-T_sk_ gradient via a reduction in T_re_ without influencing T_sk_, and CWI increased the T_re_-T_sk_ gradient via reduction in T_sk_. Additionally, CWI significantly reduced whole body sweat loss and muscle blood volume (i.e., tHb) but did not impair O_2_ utilisation as inferred from the TOI and Hb responses. The main findings from study 2 showed that CWI increased MPO and total work output during 60 min of cycling regulated at RPE 15 when compared with CON only. Additionally, both precooling strategies improved thermal sensation compared with CON, with a longer and larger effect observed following CWI. These changes occurred at similar HR response, muscle O_2_ utilisation (TOI and Hb) and skin PU between conditions. The LSR data from both studies showed that CWI and ICE delayed sweating by 1–5 min relative to CON. Furthermore, CWI resulted in a higher T_re_ threshold for sweating whereas ICE resulted in a lower T_re_ threshold for the effector response; however, sweating occurred at similar T_b_ between conditions.

ICE decreased T_re_ by ~0.3°C while CWI significantly decreased T_sk_ compared with CON and ICE in both studies (Figs 1 and 3). In study 2, the higher work output observed during the CWI trials was in agreement with the importance of T_sk_ as controller of thermoregulatory behavior during exercise in the heat [4, 29]. Notably, manipulating T_sk_ via different ambient temperatures (18°C, 26°C, 34°C, and 42°C) did not influence T_re_ but resulted in impaired exercise capacity at a higher T_sk_ [29]. Similarly, self-selected work rate during 60 min of cycling has also been shown to parallel the changes in T_sk_ rather than T_re_ [4]. Indeed, T_sk_ appeared to have a greater influence on thermoregulatory behavior than T_re_ since the reduction in T_re_ via ICE did not influence total work output when compared with CON in study 2. Nevertheless, this current observation should be viewed with the understanding that T_re_ as an index of inner body temperature is confounded by its delayed response.

T_sk_ and thermal perception have been identified as independent controllers for thermoregulatory behavior [1]. However, study 2 showed that decreased thermal sensation in ICE did not have any beneficial effect on the work capacity when RPE was clamped at 15. Our findings also contradicted previous works which demonstrated that L-Menthol mouth rinse and face cooling improved thermal perception and increased time to exhaustion during a RPE clamp exercise without concomitant changes in the physical thermal state (i.e., T_sk_ and T_re_) [3, 5]. It should be noted that maximum exercise duration within these two studies was less than 30 min, which paralleled the time course of changes in thermal sensation between ICE and CON in study 2 (Fig 3D). Taken together, the current findings do not refute the importance of thermal perception as controller of thermoregulatory behavior but suggest that the psychophysiological effect of ICE and other cooling methods are most important during shorter trials. A larger heat storage capacity conferred by ICE as a precooling strategy is negated by a lower evaporative heat loss [30], which helps to explain the lack of a clear ergogenic effect of ICE on endurance performance herein and within the literature [6]. In contrast, CWI significantly improved thermal sensation when compared with CON and ICE for up to 35–40 min during the RPE clamp exercise (study 2), supporting the importance of T_sk_ and thermal perception for mediating thermoregulatory behavior.

In line with our initial hypothesis, ICE decreased the T_re_-T_sk_ gradient whereas CWI significantly increased the T_re_-T_sk_ gradient (Figs 1 and 3). A high skin temperature (>35°C) and a concerted decrease in the T_re_-T_sk_ gradient increase skin blood flow demand for heat dissipation [31]. CWI may alleviate cardiovascular strain by reducing reliance on cutaneous vasodilation for heat loss [7]. Indeed, study 1 showed that CWI resulted in a marginal decrease of ~5 bpm in mean HR compared with CON during the exercise, consistent with others who observed a transient decrease in the HR response to exercise following CWI at 17.7°C x 30 min [32]. However, a narrower T_re_-T_sk_ gradient during early exercise stages following ICE did not affect the HR response compared with CON, which may be due to a greater increase in T_re_, causing the T_re_-T_sk_ gradient in the ICE trials to increase rapidly to levels similar to the CON trials. Another interesting observation was that although ICE and CWI decreased skin PU before the exercise, there were no differences between all conditions during the RPE clamp exercise in study 2 (Fig 4). For the ICE trials, this was concurrent with a higher T_sk_ during the first 5 min of exercise compared with CON (Fig 3), which may be related to the narrow temperature gradient between the environment, core body and skin [33], and attenuated evaporative heat loss secondary to a delayed sweating onset (Table 2). The cooling effect of CWI on the T_sk_ and T_re_-T_sk_ gradient was observed for up to 15–20 min during the exercise (Fig 3), thereby maintaining the convective heat flux from the core body to the skin during early exercise phases. However, as the environmental temperature was much higher than T_sk_ following CWI, convective heat flux to the environment was most likely reversed until T_sk_ approximated the environmental temperature, which may help to explain the lack of differences between conditions in skin PU during the exercise.

ICE purportedly improves gross efficiency and has a glycogen sparing effect related to the core body cooling effect [34]. However, no differences in the NIRS parameters were observed in both studies (Figs 2 and 4), which suggested that ICE did not affect muscle metabolism. Conversely, CWI decreased muscle blood volume and limited O_2_ delivery to locomotive muscle during the precooling period and early exercise stages in both studies. It has been previously demonstrated that 5–15 min of CWI at 10°C impaired muscle blood volume and local tissue oxygenation [9, 10], resulting in greater anaerobic contribution during intermittent high-intensity exercise in temperate environments [10]. Contrary to these earlier studies, the present study provided evidence that CWI-induced decrease in muscle blood volume did not adversely affect O_2_ utilisation during the steady state exercise (study 1) or the RPE clamp exercise (study 2) as inferred from the changes in the TOI and Hb, attributable to a more thermally comfortable CWI at 22°C versus CWI at 10°C. Importantly, a higher work output was achieved during the CWI trials at muscle O_2_ utilisation and HR response similar to CON, consistent with the propositions that relative exercise intensity was maintained secondary to alleviated cardiovascular strain [35], and that individuals paced themselves such that metabolic homeostasis was maintained [22]. It is also possible that a certain magnitude of the increase in muscle blood volume during exercise in the no-cooling trials is related to elevated heat stress and not to metabolic demand *per se*. In support, it has been shown that heat stress at rest and during exercise increases blood flow to the muscle vasculature related to a direct thermal response, an increase in arterial plasma adenosine triphosphate and/or modulation by the muscle sympathetic vasoconstrictor activity [36]. Regardless, the present results are delimited to submaximal exercise, and do not refute the possible effects that CWI may have on muscle metabolic inertia during high-intensity exercise [10].

Both CWI and ICE delayed sweating response in terms of exercise time, in agreement with previous precooling studies [32, 37, 38]. We found that sweat recruitment occurred at a lower T_re_ in ICE and at a higher T_re_ in CWI compared with CON (Table 2), whereas others observed unchanged T_re_ sweating threshold [32], or lower T_b_ sweating threshold determined from weighted T_sk_ and esophageal temperature [39] after cooling. While the disparity may be due to the different core temperature indexes or different cooling techniques (cold air versus CWI), it is more likely attributable to the influence that skin and core temperatures have on the sweat response. Indeed, sweating occurred at similar T_b_ for all conditions and the differences in sweat gain between conditions were attenuated when plotted against T_b_ (Table 2). Because ICE primarily activates the sudomotor response via the abdominal thermoreceptors [16], it is not surprising that the sweat gain against T_b_ and T_re_ are similar, and the difference in sweat sensitivity between CON and ICE did not reach statistical significance. Conversely, CWI modifies the sweat responses via the cutaneous afferent signals and the nitric oxide pathway [14]. Because T_re_ was similar between CWI and CON in studies 1 and 2, we suggest that the higher sweat gain following CWI reflects the rapid increase in T_sk_ during the exercise. Taken together, our data demonstrate that precooling attenuates the thermal afferent signals such that the efferent signals start firing when a specific T_b_ threshold is attained, but CWI modifies the efferent signals by the changes in T_sk_.

### Limitations

The NIRS parameters derived using the modified Beer-Lambert method are known to be affected by skin blood flow, whereas TOI is based on the spatially-resolved spectroscopy and has been shown to be affected by a lesser degree [28, 40]. It is important to note that the aforementioned studies examined the NIRS signals during heat stress at rest or a brief bout of single joint exercise. In contrast, skin blood flow has been shown to have a lesser influence on the NIRS signal during CWI [41]. If the cutaneous interference did contribute to the NIRS signal, it seems logical to have both parameters change similarly by cooling or heating. Moreover, skin and muscle blood flow increase drastically during prolonged exercise in the heat, and muscle blood flow has been shown to increase during local heating [42]. Therefore, while the contribution of skin blood flow to the NIRS signals during exercise cannot be dismissed, it remains challenging to distinguish between thermoregulatory and metabolic demands from the cutaneous interference.

CVC is often expressed as percentage of maximum cutaneous vasodilation achieved by local heating and/or administration of sodium nitroprusside. However, the reliability of baseline CVC, expressed as absolute values or relative to maximum CVC, remains poor with coefficients of variation ranged from 25–30% [43]. Improved reliability has been observed as CVC increases during local heating [43] or during exercise [44]. Moreover, our pilot observation showed that it took more than 40 min to achieve a plateau in CVC after the exercise. As such, skin PU data are expressed in absolute values.

The difference in power output between CON and CWI in study 2 is considered as small effect (Cohen’s *d* = 0.42, mean difference of 8 W). The modest beneficial effect from CWI may be related to the training status of the present cohort (${\dot{V}O}_{2\mathrm{peak}}$ <55 mL.kg^-1^.min^-1^), as well-trained athletes have been shown to have a greater beneficial effect from precooling maneuvers [8]. Additionally, the present results are based on a male sample. Future research should explore the possible gender effect on precooling and the resultant physiological responses, as it has been shown that females have a lower evaporative heat loss and blunted sweating thermosensitivity than males during exercise at a fixed rate of metabolic heat production after controlling for menstrual cycle, body mass, and ${\dot{V}O}_{2\mathrm{peak}}$ relative to lean muscle mass [45].

### Conclusions

To conclude, significant skin cooing by CWI resulted in a greater reduction in thermal sensation and improved thermoregulatory behavior (i.e., higher MPO and total work output). Additionally, although CWI decreased muscle blood volume during early exercise stages, it did not limit muscle oxygenation during steady state exercise or RPE clamp exercise in the heat. Conversely, a reduction of ~0.3°C in T_re_ by ICE decreased thermal sensation but did not confer any ergogenic effect on the thermoregulatory behavior during 60 min of exercise. As such, the duration of events should be taken into consideration when planning for cooling strategies prior to exercise in hot conditions. ICE and CWI delayed sweat recruitment in terms of exercise time and attenuated the thermal efferent signals until a specific T_b_ threshold was attained, but the efferent signals were modified by the changes in T_sk_ in CWI.

## Supporting information

S1 Table Rectal temperature (T_re_) response during 60 min of cycling at fixed intensity following 30 min of precooling (study 1). CON, control; CWI, cold water immersion, ICE, ice slushy ingestion.
(PDF)

S2 Table Rectal-to-skin temperature (T_re_-T_sk_) gradient response during 60 min of cycling at fixed intensity following 30 min of precooling (study 1). CON, control; CWI, cold water immersion, ICE, ice slushy ingestion.
(PDF)

S3 Table Rectal temperature (T_re_) response during 60 min of cycling at RPE 15 following 30 min of precooling (study 2). CON, control; CWI, cold water immersion, ICE, ice slushy ingestion.
(PDF)

S4 Table Rectal-to-skin temperature (T_re_-T_sk_) gradient response during 60 min of cycling at RPE 15 following 30 min of precooling (study 2). CON, control; CWI, cold water immersion, ICE, ice slushy ingestion.
(PDF)

S5 Table Effect sizes (Cohen’s *d*) calculated for the mean physiological and perceptual responses, rectal temperature (T_re_) and weighted mean body temperature (T_b_) at the onset of sweating and sweat sensitivity during steady state exercise (study 1) and during the RPE clamp exercise (study 2). CON, control; CWI, cold water immersion, ICE, ice slushy ingestion.
(PDF)

S1 Dataset Data underlying the findings reported in the present manuscript. CON, control; CWI, cold water immersion, ICE, ice slushy ingestion.
(XLSX)
