# Supplementary material for: Effect of ice slushy ingestion and cold water immersion on thermoregulatory behavior
Source: PLoS One. 2019 Feb 27;14(2):e0212966. doi: 10.1371/journal.pone.0212966 (PMC6392407; doi:10.1371/journal.pone.0212966)
Supplement: S1 Table — CON, control; CWI, cold water immersion, ICE, ice slushy ingestion. (PDF) [file pone.0212966.s001.pdf]

S1 Table. Rectal temperature (T_{re}) response during 60 min of cycling at fixed intensity following 30 min of precooling (study 1). CON, control; CWI, cold water immersion, ICE, ice slushy ingestion.

	CON		CWI		ICE	
**Precooling**	***n***	**T_{re} (°C)**	***n***	**T_{re} (°C)**	***n***	**T_{re} (°C)**
0 min	10	37.3 ± 0.3	10	37.3 ± 0.1	10	37.3 ± 0.2
30 min	10	37.3 ± 0.3	10	37.4 ± 0.1	10	37.1 ± 0.3
**Exercise**						
0 min	10	37.3 ± 0.3	10	37.4 ± 0.1	10	37.0 ± 0.3
5 min	10	37.4 ± 0.2	10	37.4 ± 0.2	10	37.1 ± 0.2
10 min	10	37.5 ± 0.2	10	37.5 ± 0.2	10	37.2 ± 0.2
15 min	10	37.6 ± 0.2	10	37.6 ± 0.2	9	37.4 ± 0.3
20 min	10	37.7 ± 0.3	9	37.7 ± 0.3	9	37.6 ± 0.3
25 min	10	37.8 ± 0.3	9	37.8 ± 0.3	9	37.7 ± 0.3
30 min	10	37.9 ± 0.3	9	37.9 ± 0.4	9	37.9 ± 0.4
35 min	10	38.0 ± 0.3	9	38.0 ± 0.4	9	38.0 ± 0.4
40 min	10	38.1 ± 0.4	9	38.1 ± 0.4	9	38.2 ± 0.5
45 min	9	38.3 ± 0.4	8	38.2 ± 0.5	9	38.3 ± 0.5
50 min	9	38.4 ± 0.5	8	38.3 ± 0.6	9	38.4 ± 0.5
55 min	9	38.5 ± 0.5	8	38.5 ± 0.6	8	38.5 ± 0.6
60 min	9	38.6 ± 0.6	8	38.6 ± 0.6	9	38.7 ± 0.7
