# Supplementary material for: Effect of ice slushy ingestion and cold water immersion on thermoregulatory behavior
Source: PLoS One. 2019 Feb 27;14(2):e0212966. doi: 10.1371/journal.pone.0212966 (PMC6392407; doi:10.1371/journal.pone.0212966)
Supplement: S2 Table — CON, control; CWI, cold water immersion, ICE, ice slushy ingestion. (PDF) [file pone.0212966.s002.pdf]

S2 Table. Rectal-to-skin temperature (T_{re}-T_{sk}) gradient response during 60 min of cycling at fixed intensity following 30 min of precooling (study 1). CON, control; CWI, cold water immersion, ICE, ice slushy ingestion.

	CON		CWI		ICE	
**Precooling**	***n***	**T_{re}-T_{sk} (°C)**	***n***	**T_{re}-T_{sk} (°C)**	***n***	**T_{re}-T_{sk} (°C)**
0 min	10	2.9 ± 0.5	10	2.6 ± 0.4	10	2.7 ± 0.5
30 min	10	2.6 ± 0.5	10	5.4 ± 0.4	10	2.2 ± 0.4
**Exercise**						
0 min	10	2.7 ± 0.5	10	4 ± 0.3	10	2.1 ± 0.3
5 mn	10	2.8 ± 0.5	10	3.6 ± 0.5	10	2.3 ± 0.3
10 min	10	2.4 ± 0.5	10	3.2 ± 0.5	10	2.3 ± 0.5
15 min	10	2.4 ± 0.5	10	2.9 ± 0.6	9	2.3 ± 0.5
20 min	10	2.4 ± 0.5	9	2.7 ± 0.6	9	2.3 ± 0.5
25 min	10	2.5 ± 0.6	9	2.8 ± 0.6	9	2.4 ± 0.6
30 min	10	2.6 ± 0.6	9	2.8 ± 0.6	9	2.6 ± 0.6
35 min	10	2.7 ± 0.6	9	2.9 ± 0.6	9	2.7 ± 0.6
40 min	10	2.8 ± 0.6	9	3 ± 0.6	9	2.8 ± 0.6
45 min	9	2.8 ± 0.6	8	3.2 ± 0.6	9	2.9 ± 0.7
50 min	9	2.8 ± 0.6	8	3.3 ± 0.7	9	2.9 ± 0.6
55 min	9	2.9 ± 0.7	8	3.3 ± 0.7	8	3.1 ± 0.7
60 min	9	2.9 ± 0.7	8	3.4 ± 0.7	9	3.1 ± 0.7
