# Supplementary material for: Effect of ice slushy ingestion and cold water immersion on thermoregulatory behavior
Source: PLoS One. 2019 Feb 27;14(2):e0212966. doi: 10.1371/journal.pone.0212966 (PMC6392407; doi:10.1371/journal.pone.0212966)
Supplement: S3 Table — CON, control; CWI, cold water immersion, ICE, ice slushy ingestion. (PDF) [file pone.0212966.s003.pdf]

S3 Table. Rectal temperature (T_{re}) response during 60 min of cycling at RPE 15 following 30 min of precooling (study 2). CON, control; CWI, cold water immersion, ICE, ice slushy ingestion.

	**CON**		**CWI**		**ICE**	
**Precooling**	***n***	**T_{re} (°C)**	***n***	**T_{re} (°C)**	***n***	**T_{re} (°C)**
0 min	11	37.3 ± 0.5	11	37.2 ± 0.3	11	37.2 ± 0.3
30 min	11	37.3 ± 0.4	11	37.4 ± 0.3	11	37.0 ± 0.4
**Exercise**						
0 min	11	37.3 ± 0.4	11	37.3 ± 0.3	11	37.0 ± 0.4
5 mn	11	37.3 ± 0.4	11	37.3 ± 0.3	11	37.0 ± 0.4
10 min	11	37.4 ± 0.4	11	37.4 ± 0.3	11	37.1 ± 0.4
15 min	11	37.6 ± 0.4	11	37.5 ± 0.3	11	37.3 ± 0.4
20 min	11	37.7 ± 0.4	11	37.6 ± 0.3	11	37.5 ± 0.4
25 min	11	37.9 ± 0.5	11	37.8 ± 0.4	11	37.7 ± 0.4
30 min	11	38.0 ± 0.5	10	37.9 ± 0.4	11	37.9 ± 0.4
35 min	11	38.2 ± 0.5	10	38.0 ± 0.4	11	38.0 ± 0.4
40 min	11	38.3 ± 0.5	10	38.1 ± 0.4	11	38.2 ± 0.4
45 min	11	38.4 ± 0.5	11	38.3 ± 0.4	11	38.3 ± 0.4
50 min	11	38.5 ± 0.5	10	38.3 ± 0.4	10	38.4 ± 0.5
55 min	11	38.5 ± 0.5	10	38.4 ± 0.5	10	38.6 ± 0.5
60 min	11	38.6 ± 0.5	11	38.6 ± 0.5	10	38.6 ± 0.4
