# Supplementary material for: Effect of ice slushy ingestion and cold water immersion on thermoregulatory behavior
Source: PLoS One. 2019 Feb 27;14(2):e0212966. doi: 10.1371/journal.pone.0212966 (PMC6392407; doi:10.1371/journal.pone.0212966)
Supplement: S4 Table — CON, control; CWI, cold water immersion, ICE, ice slushy ingestion. (PDF) [file pone.0212966.s004.pdf]

S4 Table. Rectal-to-skin temperature (T_{re}-T_{sk}) gradient response during 60 min of cycling at RPE 15 following 30 min of precooling (study 2). CON, control; CWI, cold water immersion, ICE, ice slushy ingestion.

	CON		CWI		ICE	
**Precooling**	***n***	**T_{re}-T_{sk} (°C)**	***n***	**T_{re}-T_{sk} (°C)**	***n***	**T_{re}-T_{sk} (°C)**
0 min	11	2.7 ± 0.5	11	2.5 ± 0.4	11	2.6 ± 0.3
30 min	11	2.6 ± 0.6	11	5.6 ± 0.5	11	2.0 ± 0.3
**Exercise**						
0 min	11	2.8 ± 0.7	11	4.0 ± 0.6	11	1.8 ± 0.3
5 mn	11	2.9 ± 0.7	11	3.7 ± 0.9	11	2.0 ± 0.4
10 min	11	2.4 ± 0.5	11	3.3 ± 0.9	11	2.0 ± 0.2
15 min	11	2.3 ± 0.4	11	2.9 ± 0.6	11	2.0 ± 0.3
20 min	11	2.4 ± 0.4	11	2.7 ± 0.5	11	2.2 ± 0.3
25 min	11	2.5 ± 0.4	11	2.8 ± 0.4	11	2.3 ± 0.4
30 min	11	2.7 ± 0.3	10	2.9 ± 0.4	11	2.5 ± 0.5
35 min	11	2.7 ± 0.4	10	2.9 ± 0.4	11	2.7 ± 0.5
40 min	11	2.8 ± 0.4	10	3.0 ± 0.4	11	2.8 ± 0.5
45 min	11	2.9 ± 0.4	11	3.2 ± 0.3	11	3.0 ± 0.5
50 min	11	3.0 ± 0.4	10	3.2 ± 0.3	10	3.2 ± 0.6
55 min	11	3.1 ± 0.4	10	3.3 ± 0.4	10	3.4 ± 0.7
60 min	11	3.1 ± 0.5	11	3.4 ± 0.5	10	3.3 ± 0.5
