# Supplementary material for: Effect of ice slushy ingestion and cold water immersion on thermoregulatory behavior
Source: PLoS One. 2019 Feb 27;14(2):e0212966. doi: 10.1371/journal.pone.0212966 (PMC6392407; doi:10.1371/journal.pone.0212966)
Supplement: S5 Table — CON, control; CWI, cold water immersion, ICE, ice slushy ingestion. (PDF) [file pone.0212966.s005.pdf]

S5 Table. Effect sizes (Cohen's *d*) calculated for the mean physiological and perceptual responses, rectal temperature (T_{re}) and weighted mean body temperature (T_b) at the onset of sweating and sweat sensitivity during steady state exercise (study 1) and during the RPE clamp exercise (study 2). CON, control; CWI, cold water immersion, ICE, ice slushy ingestion.

					Cohen's *d*		
**Study 1:**	***n***	**CON**	**CWI**	**ICE**	**CON vs CWI**	**CON vs ICE**	**CWI vs ICE**
Whole body sweat loss (mL)	11	1244 ± 374	1064 ± 343	1128 ± 329	0.50	0.33	0.19
Thermal sensation (AU)	11	6 ± 1.1	5.7 ± 0.6	5.8 ± 0.7	0.34	0.22	0.15
RPE (AU)	11	13.5 ± 1.9	13.4 ± 1.8	13.4 ± 1.6	0.05	0.06	0.00
Heart rate (beats·min^{-1})	11	154 ± 14	149 ± 15	153 ± 14	0.34	0.07	0.28
$T_{re}\Delta$ (°C)	8	1.3 ± 0.5	1.2 ± 0.6	1.6 ± 0.6	0.18	0.54	0.67
LSR_{arm}	11	1.25 ± 0.75	1.1 ± 0.53	1.14 ± 0.44	0.23	0.18	0.08
LSR_{th}	11	0.71 ± 0.46	0.74 ± 0.36	0.69 ± 0.35	0.07	0.05	0.14
**Study 2:**							
Mean power output (W)	11	130 ± 20	138 ± 18	129 ± 25	0.42	0.04	0.41
Total work output (kJ)	11	470 ± 74	498 ± 65	464 ± 90	0.40	0.07	0.43
Whole body sweat loss (mL)	11	1394 ± 381	1239 ± 367	1396 ± 119	0.41	0.01	0.58
Heart rate (beats·min^{-1})	11	144 ± 20	141 ± 14	144 ± 15	0.17	0.00	0.21
$T_{re}\Delta$ (°C)	11	1.4 ± 0.5	1.2 ± 0.6	1.7 ± 0.5	0.36	0.60	0.91
LSR_{arm}	11	1.21 ± 0.35	1.21 ± 0.53	1.2 ± 0.6	0.00	0.02	0.02
LSR_{th}	11	0.63 ± 0.19	0.68 ± 0.29	0.66 ± 0.27	0.20	0.13	0.07
**T_{re} and T_b at the sweat threshold and sweat sensitivity:**							
Onset of sweating (min)	19	1.8 ± 1.8	6.4 ± 2.2	2.7 ± 1.6	2.29	0.53	1.92
T_{re} sweat threshold (°C)	19	37 ± 0.3	37.1 ± 0.2	36.8 ± 0.3	0.39	0.67	1.18
T_b sweat threshold (°C)	19	36.5 ± 0.3	36.5 ± 0.3	36.5 ± 0.4	0.00	0.00	0.00
T_{re} sweat sensitivity (mg·cm^{-2}·min^{-1}·°C^{-1})	19	1.51 ± 0.61	1.89 ± 0.82	1.42 ± 0.68	0.53	0.14	0.62
T_b sweat sensitivity (mg·cm^{-2}·min^{-1}·°C^{-1})	19	1.18 ± 0.42	1.36 ± 0.55	1.39 ± 0.63	0.37	0.39	0.05
